# Efficacy and safety of needle-free jet injector-assisted intralesional treatments in dermatology—a systematic review

**DOI:** 10.1007/s13346-023-01295-x

**Published:** 2023-03-08

**Authors:** Vazula Zulfra Bekkers, Liora Bik, Johanna Catharina van Huijstee, Albert Wolkerstorfer, Errol Prospero Prens, Martijn Bastiaan Adriaan van Doorn

**Affiliations:** 1 Department of Dermatology, Erasmus Medical Center, Rotterdam, the Netherlands; 2 Department of Dermatology, Amsterdam University Medical Center, Location AMC, Amsterdam, the Netherlands

**Keywords:** Jet injection, Needle-free injection, Efficacy, Safety, Dermatology, Systematic review

## Abstract

Needle-free jet injectors are used for the intralesional treatment of various dermatological indications. However, a systematic review that evaluates the efficacy and safety of these treatments has not been published. The objectives of this study are to evaluate the efficacy and safety of needle-free jet injections for dermatological indications and to provide evidence-based treatment recommendations. An electronic literature search was conducted in April 2022. Two reviewers independently selected studies based on predefined criteria and performed a methodological quality assessment using the Cochrane Collaborations risk-of-bias 2.0 assessment tool and Newcastle–Ottawa Scale. Thirty-seven articles were included, involving 1911 participants. Dermatological indications included scars, alopecia areata, hyperhidrosis, nail diseases, non-melanoma skin cancer, common warts, local anesthesia, and aesthetic indications. Keloids and other types of scars (hypertrophic, atrophic, and burn scars) were investigated most frequently (*n* = 7). The included studies reported favorable efficacy and safety outcomes for intralesional jet injector-assisted treatment with triamcinolone acetonide/hexacetonide, 5-fluorouracil, bleomycin, or hyaluronic acid. Two high-quality studies showed good efficacy and tolerability of intralesional jet injections with a combination of 5-fluorouracil and triamcinolone acetonide in hypertrophic scars and with saline in boxcar and rolling acne scars. No serious adverse reactions and good tolerability were reported in the included studies. Overall, the methodological quality of the included studies was low. Limited evidence suggests that needle-free jet injector-assisted intralesional treatment is efficacious and safe for hypertrophic and atrophic acne scars. More well-powered RCTs investigating the efficacy and safety of jet injector treatment in dermatology are warranted to make further evidence-based recommendations.

**Graphical Abstract:**

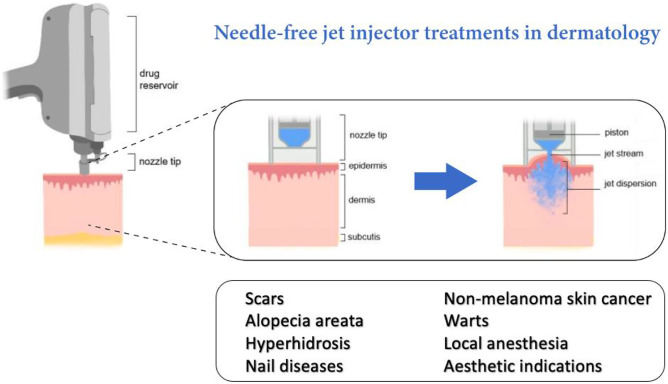

## Introduction

Intradermal drug delivery has many advantages over other routes of administration, especially high bioavailability in the skin [1, 2]. Over the past decades, a variety of needle-free devices that enable intradermal drug delivery has been developed, including fractional ablative lasers, iontophoresis, sonophoresis, and various types of mechanical and energy-based jet injectors [3–5].

Jet injectors are commonly used for the intralesional treatment of several dermatological conditions such as keloids, hypertrophic scars, and recalcitrant viral warts [6, 7]. Traditional mechanical jet injectors act with a fixed pressure predetermined by spring size [8]. Innovative electronically controlled pneumatic jet injectors are devices in which volume and pressure can be controlled by accelerated and compressed gas as pressure source, which dispense fluids into the skin [7, 9]. Other types of jet injectors are controlled by Lorentz or piezoelectric actuators, lasers, and shockwaves to pressurize the injected drug [10].

In contemporary healthcare, we are moving towards more patient-centered care. It is important to improve patient comfort and avoid physical or psychological harm as much as possible. According to a previous study, 63% of children and 24% of the adult population in the USA fear needles [11]. This is one of the reasons why jet injectors can be a viable alternative for conventional needles.

Needle-free jet injectors can be an attractive alternative for hypodermic needles for patients experiencing needle phobia, minimize treatment-related pain, and are free of risk for needlestick injuries and cross-contamination. Additionally, jet injectors enable accurate and reproducible dermal delivery of liquid drugs and disperse the drug more evenly in the skin than conventional needle injections [7, 9, 12, 13].

At present, there are a few overviews and narrative reviews describing the use of jet injector-assisted intralesional treatment for different dermatological indications [7, 10, 12, 14]. However, a systematic and critical review that evaluates the efficacy and safety of jet injector-assisted intralesional treatment in dermatology is lacking. In this review, we aimed to systematically review and evaluate the quality of clinical evidence for intralesional treatment of dermatological indications using needle-free jet injector systems and provide evidence-based recommendations for clinical practice.

## Materials and methods

A literature search was conducted in April 2022 using Embase, MEDLINE ALL Ovid, Web of Science, and Cochrane Central Register of Controlled Trials databases, to identify relevant publications. This systematic review was registered in the PROSPERO (CRD42021258278) and followed the Preferred Reporting Items for the PRISMA 2020 checklist [15].

Studies were included if they were human studies, written in English, published from inception to April 2022, randomized controlled trials (RCTs), controlled clinical trials (CCTs), prospective or retrospective cohort studies, and case series and included patients of all ages with dermatological indications eligible for intralesional treatment using needle-free jet injectors. Exclusion criteria included studies with fewer than 10 patients and intramuscular or subcutaneous drug delivery.

Selection of the articles, standardized data extraction, and methodological quality assessment of the included studies were performed independently by two authors (V.B. and J.V.H.). Articles were screened based on title and abstract. The primary outcome measure was efficacy, and the secondary outcome measure was safety. For data extraction, we converted pressure settings, total injection volume, and drug concentration to psi, ml, and mg/ml, respectively. If possible, efficacy measures were simplified to percentages in terms of clinical response compared to baseline. Methodological quality was assessed using the Cochrane Collaborations risk-of-bias 2.0 tool (ROB 2.0) for RCTs and CCTs, and the Newcastle–Ottawa Scale (NOS) for cohort studies and case series [16–19]. Final selection of the articles was based on screening of full texts. Discrepancies between reviewers were discussed and resolved by consensus and involved a third author (L.B.) if necessary. Illustrations of the methodological quality assessments were created using Robvis [17].

## Results

**Fig. 1:**
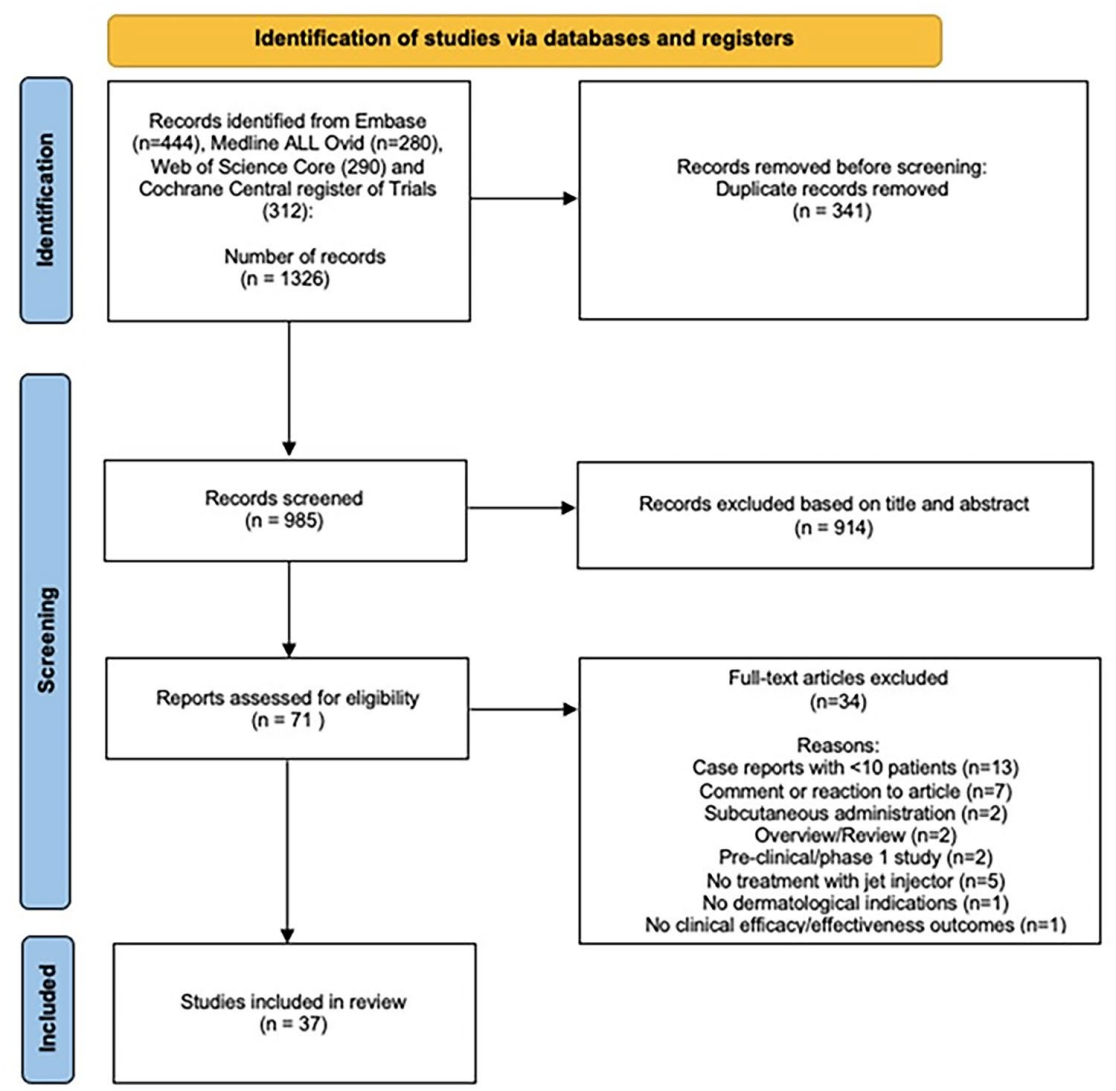
Study flow diagram of exclusion process resulting in 37 included studies

Our literature search identified 1326 records. Duplicates were removed. Based on title and abstract, 985 articles were screened. Full texts of 71 articles were assessed for eligibility of which 37 studies were selected with a total of 1911 participants (Fig. 1). The included studies comprised 6 RCTs, 6 CCTs, 16 prospective cohorts, 5 retrospective cohorts, and 4 case series. The studies investigated needle-free jet injector-assisted intralesional treatments for atrophic and hypertrophic scars, keloids, alopecia areata, hyperhidrosis, nail diseases (psoriasis, lichen planus, and idiopathic onycholysis), non-melanoma skin cancer (basal cell carcinoma (BCC), squamous cell carcinoma (SCC), Bowen’s disease, and Paget’s disease), common warts, granuloma annulare, lichen simplex chronicus, psoriasis, seborrheic dermatitis, aesthetic indications (wrinkles, rejuvenation, rhytides, facelift), and local anesthesia.

### Scars and keloids

Seven studies, investigated jet injections to treat various scar types (Table 1) [20–28]. Compared to baseline, spring-loaded jet injections with triamcinolone acetonide (TCA) and silicone sheets showed significant scar thickness reduction in hypertrophic scars, while silicone sheets alone did not (3–5 treatments; *p* < 0.05; *p* > 0.05) [21]. Moreover, pneumatic jet injector-assisted treatment with a mixture of hyaluronic acid and hypertonic glucose led to a reduction in mean scar volume of 0.4 mm^3^ compared to the untreated side in atrophic facial acne scars (single treatment; *p* < 0.05) [23]. Spring-loaded jet injections with bleomycin in keloids and hypertrophic scars led to reduced pain and pruritus with respectively 88% and 89% (2–6 treatments; no comparative intervention; no statistical analyses reported) [26]. Furthermore, pneumatic jet injections with 5-fluorouracil (5-FU) diluted in corticosteroids (TCA or methylprednisolone acetate) and lidocaine led to a significant reduction of pain and pruritus in patients with keloids, with respectively 69% and 79% compared to baseline (7 treatments; no comparative intervention; *p* < 0.01; *p* < 0.05) [27]. Pneumatic jet injections of hypertonic glucose resulted in a mean Global Aesthetic Improvement Scale (GAIS) of 2.3 ± 0.8 in atrophic scars, striae, and wrinkles compared to baseline (1–5 treatments; no comparative intervention; no statistical analyses) [24]. In comparison, jet injections with non-crosslinked and crosslinked hyaluronic acid injections in acne and hypertrophic scars resulted in overall GAIS of 1.9 and 1.8 respectively (mean 2.5 treatments; no statistical analyses) [25]. Jet injections (unknown injector type) with triamcinolone hexacetonide resulted in “good,” “acceptable,” and “negative” results in respectively 68.2%, 15.9%, and 15.9% of children with burn scars (1–4 series, no comparative intervention; no statistical analyses) [28].

**Table 1:** Characteristics and summary of results of included studies using needle-free jet injectors in scars and keloids, alopecia areata, hyperhidrosis, nail diseases, non-melanoma skin cancer, and warts

**First author, year of publication**	**Dermatological indication**	**Study design**	**No. of patients (+ lesions)**	**Skin type**	**Type jet injector (brand) + (pressure)**	**Pressure in study**	**Fluid**
Erlendsson, 2022	Hypertrophic scars	RCT	20 (?)	I–V	Pneumatic (Enerjet 2.0) (A)	30.4–52.2 psi	A: 5-FU + TCA
Alshehari, 2015	Hypertrophic scars	RCT	30 (?)	NR	Spring-loaded (Dermojet) (F)	1420 psi	A: TCA + silicone sheet
Pravangsuk, 2021	Atrophic acne scars (boxcar and rolling)	RCT	18 (108)	III–IV	Pneumatic (Innojector) (A)	Unclear (levels 2–3)	A: SAL
Kim, 2019	Atrophic acne scars (boxcar, rolling and icepick)	Prospective cohort	10 (13)	II–IV	A: Pneumatic (Airjet) (A)	Unclear (50% of total)	A: HA in hypertonic glucose
Kim, 2017	Atrophic scars (post acne, carbuncle, furuncle), striae and wrinkles	Prospective cohort	13 (13)	III–IV	Pneumatic (SheMax) (A)	52.6–67.3 psi	Hypertonic glucose
MacGillis, 2021	Scars, skin rejuvenation, striae	Retrospective cohort	115 (325)	NR	Pneumatic (Enerjet/Airgent) (A)	NR	Crosslinked HA in SAL
Saray, 2005	Keloids and hypertrophic scars	Prospective cohort	14 (15)	II–IV	Spring-loaded (MadaJet XL) (F)	1800 psi	Bleomycin in SAL
Levenberg, 2020	Keloids	Retrospective cohort	20 (38)	NR	Pneumatic (Enerjet 2.0) (A)	43.5–82.3 psi	5-FU in MA/TCA + lidocaine 2%
Grisolia, 1983	Burn scars	CS	44 (?)	NR	NR	NR	TH in SAL
Metin, 1999	Alopecia areata	CCT	35 (?)	NR	NR	NR	A: BDSP or SAL
Abell, 1973	Alopecia areata	Prospective cohort	84 (111)	NR	Spring-loaded (Port-O-Jet) (F)	NR	TCA
Mallick, 2018	Alopecia areata	CS	100 (?)	NR	Spring-loaded (Dermojet) (F)	1420 psi	TCA
Moynahan, 1965	Alopecia areata	CS	60 (60)	NR	Spring-loaded (Porton needleless injector) (F)	NR	TCA
Vadeboncoeur, 2017	Palmar hyperhidrosis	CCT	20 (40)	NR	Pneumatic (Med-Jet) (A)	140–150 psi	A: direct OnabotA in SAL
Naumann, 1999	Palmar and axillar Hyperhidrosis	CCT	20 (40)	NR	A: Spring-loaded (Dermojet) (F)	1420 psi	A: BTX-A in SAL
Kim, 2020	Axillar and palmoplantar hyperhidrosis	Prospective cohort	20 (?)	NR	SheMax (A)	29.7 psi	BoNT-A in SAL and lidocaine 2%
Vadoud-Seyedi, 2004	Plantar hyperhidrosis	Prospective cohort	10 (20)	NR	Spring-loaded (Dermojet) (F)	1420 psi	BTX-A in SAL
Nantel-Battista, 2014	Nail psoriasis	Prospective cohort	16 (16)	NR	Pneumatic (Med-Jet) (A)	130–170 psi	TCA
Peachey, 1976	Nail psoriasis	Prospective cohort	37 (37)	NR	Spring-loaded (Port-O-Jet) (F)	NR	A: TCA
Abell, 1973	Nail dystrophy	Prospective cohort	100 (693)	NR	Spring-loaded (Port-O-Jet) (F)	NR	TCA
Gong, 2016	Non-melanoma skin cancer	Prospective cohort	54 (54)	NR	Spring-loaded (INJEX) (F)**	3000 psi	5-ALA
Zhao, 2020	Non-melanoma skin cancer	Retrospective cohort	381 (381)	NR	A: Pneumatic (Airjet) (A)**	NR	A: 5-ALA
Agius, 2006	Plantar warts	Prospective cohort	47 (138)	NR	Spring-loaded (Dermojet) (F)	1420 psi	Bleomycin
Brodell, 1995	Palmar/plantar warts	CS	22 (> 49)	NR	Spring-loaded (Dermojet) (F)	1420 psi	Interferon alfa-n3

**First author, year of publication**	**Total volume per lesion each treatment**	**Concentration**	**Total no treatments and interval**	**Comparison**	**Results per patient + significance (results per lesion + significance)**	**Follow-up time**	**Adverse reactions**	**Ref**
Erlendsson, 2022	0.32–0.70 ml	5-FU: 50 mg/ml, TCA: 10 or 40 mg/ml	1	B: No treatment	NR (Total VSS decreased in 55% and 25%, resp. in A and B, with median reduction of − 1 in VSS score (0 in control; *p* = 0.09))	1 month	Severe: none Minor: punctate defects, hyperpigmentation	[20]
Alshehari, 2015	Single or multiple doses of 0.1 ml	40 mg/ml	3–5, 3 weeks	B: Silicone sheet alone	Scar thickness reduced in A and B, *p* < 0.05; *p* > 0.05. (NR)	6 months	Severe: none Minor: pain	[21]
Pravangsuk, 2021	Unclear (first two treatments shots of 0.15 ml, third treatment shot of 0.1 ml)	9 mg/ml	3, 4 weeks	B: Needle subcision	NR (Mean scar volume reduced with 11.7% and 12.0% compared to baseline, resp. in A and B, *p* < 0.001; *p* < 0.001. No statistical difference between treatments)	1 month	Severe: none Minor: bruises, scale, hyperpigmentation, hematoma, oedema, erythema and subcutaneous emphysema	[22]
Kim, 2019	NR (0.085 ml injection)	HA: 1 mg/mL, glucose: 200 mg/ml	1	B: No treatment	Mean scar volume reduced with ca. 0.4 mm3 and 0.0 mm3, resp. in A and B (*p* < 0.05). (NR)	2 months	Severe: none Minor: swelling, spot bleeding	[23]
Kim, 2017	NR (0.08–0.1 ml per injection)	200 mg/ml	1–5, 3 weeks	None	Mean GAIS 1 month after final treatment 2.3 ± 0.8. NS. (NR)	2 months	Severe: none Minor: spot bleeding, crusting, PIH	[24]
MacGillis, 2021	NR (0.05–0.75 ml per injection)	2.5 mg/mL	Mean 2.85, 12 weeks	Non-crosslinked HA	Overall GAIS score 1.78 and 1.6 resp. NCL-HA and CL-HA. NS. (NR)	> 3 months	Severe: none Minor: bruises, temporary local edema	[25]
Saray, 2005	< 3.5 ml	1.5 IU/ml	2–6, 4 weeks	None	NR. (Mean scar height, pliability, erythema, pain- and pruritus score reduced resp. 3.20 mm, 2.64 mm, 2.13 mm, 88%, 89%, p < 0.001; p < 0.001; *p* < 0.001; *p* = 0.01. No recurrences)	16–24 months	Severe: none Minor: hyperpigmentation and skin atrophy	[26]
Levenberg, 2020	0.5–10 ml	5-FU: 50 mg/ml, MA/TCA: 40 mg/mL	7, 2 weeks	None	NR. (Total VSS score decreased with 53% in all components, *p* < 0.05. Overall POSAS patient score decreased in all components from 39.54 ± 5.31 to 19.63 ± 6.30, *P* < 0.05. Pain and pruritus lessened resp. 69% and 79%, *p* < 0.05. No recurrence)	12 months	Severe: none Minor: superficial ulceration	[27]
Grisolia, 1983	NR (mass < 5 mg)	2 mg/ml	1–4 series, 1–3 weeks	None	“Good” in 68.2%, “acceptable” in 15.9%, and “negative” in 15.9%. NS. (NR)	NR	Severe: none Minor: telangiectasia, increased hair growth, subcutaneous atrophy, ulcer	[28]
Metin, 1999	NR	NR	4, 3 weeks	B: Cyclosporine A or SAL	NR. (Regrowth in 88.2% and 66.6%, resp. in BDSP and Cyclosporine A. Regrowth in 11.7% and 16.6% in resp. A and B with SAL. NS.)	NR	NR	[29 ]
Abell, 1973	Mean: 2.8 ml	5 mg/ml	3, 1–2 weeks	None	Regrowth in 86% and 62%, resp. after 6 and 12 weeks. NS. (NR)	3 months	Severe: fluctuating cortisol Minor: hemorrhage, atrophy	[30]
Mallick, 2018	NR (0.1 ml per injection)	10 mg/ml	3–4, 4 weeks	None	Regrowth in 75%. Stratification for age, gender, duration, fam. history, and size all > 0.05 (NR)	3 weeks	NR	[31]
Moynahan, 1965	< 6.0 ml	5 mg/ml	≤ . 3, 1 week	None	Regrowth in 49% and 43% of adults and children, resp. NS. (NR)	NR	Severe: a. temporalis damage Minor: bleeding	[32]
Vadeboncoeur, 2017	5 ml	20 U/ml	1	B: NPT + lidocaine, CNI + OnabotA	HDSS score reduced with 1.6 and 1.25 resp. in A and B after 1 month, *p* = 0.031. Reduction at 3 and 6 months not statistically significant. (NR)	6 months	Severe: none Minor: weakness, vasovagal symptoms, ecchymosis	[33]
Naumann, 1999	BTX-A: 50 MU, SAL: 5 ml	20 U/ml	1	B: CNI + BTX-A in SAL	Sweat production of 77.8 ± 8.4 and 72.2 ± 10.1 mg/ml at baseline, and 53.1 ± 7.8 and 18.1 ± 3.3 mg/ml post-treatment resp. in A and B, *p* < 0.05; *p* < 0.0001. (NR)	3–4 weeks	Severe: none Minor: hematoma, transient paresthesia	[34]
Kim, 2020	4.8–6.4 ml	6.25 U/ml	1	None	NR (Median HDSS reduction from 3 to 1 and from 4 to 1, resp. axillar and palmoplantar, *p* < 0.001; *p* < 0.001)	1 month	Severe: none Minor: subcorneal blisters	[35]
Vadoud-Seyedi, 2004	UBTX-A: 50 U, SAL: 5 ml	NR	1	None	After 5 months 70% was free of symptoms. NS. (NR)	8 months	Severe: none Minor: localized hematoma	[36]
Nantel-Battista, 2014	Ca 0.07 ml	8 mg/mL	4, 4 ± 1 weeks	None	Mean baseline NAPSI score was 6.5, mean final NAPSI score was 2.8, *p* = 0.0007. (NR)	12 months	Severe: none Minor: spot bleeding	[37]
Peachey, 1976	0.1–0.3 ml	5 mg/ml	A: 3, 4–6 weeks	B: TCA 3–9 treatments, intervals 2–4 weeks. B: TH, 3–4 treatments, intervals 5–7 weeks	Study ended premature due poor results. Improvement in 90% and 26% in resp. nail-matrix and nail-bed and/or hyponychial. NS. (NR)	1 month	Severe: none Minor: pain and atrophy	[38]
Abell, 1973	0.1–0.4 ml	5 mg/ml	At least 3, 2–10 weeks	None	Matrix improvement in matrix psoriasis, combined psoriasis, lichen planus in resp. 84%, 95%, and 73%. Onycholysis Improvement in combined psoriasis, psoriatic and idiopathic onycholysis in resp. 70%, 50%, and 47%. Overall, 42% relapsed. NS. (NR)	24 months	Severe: none Minor: hemorrhage, atrophy, penetration of the nail plate	[30]
Gong, 2016	0.4 ml	200 mg/ml	6, 2 weeks	None	CR of 81% and PR of 13%. Recurrence rate of 9% at follow-up. NS. (NR)	12 months	Severe: none Minor: swelling, rash, hyperpigmentation	[40]
Zhao, 2020	0.5 ml	200 mg/ml	6, 1–2 weeks	B: CNI + 5-ALA in SAL C: BPT + 5-ALA in SAL	CR 77%, 65%, 66% resp. in NPT, CNI, and BPT. Recurrence rate of 4% at follow-up, *p* = 0.012. (NR)	6 months	Severe: none Minor: swelling, rash, burning, itching, hyperpigmentation, headache, chills, puffy eyelids	[41]
Agius, 2006	Mean of 1–3 ml	1 U/ml	5, 5 weeks	None	NR. (CR was 51.5%, 60.1%, 73.9%, 77.5%, and 77.5% after resp. first, second, third, fourth, and fifth treatments. NS)	5 weeks	Severe: cellulitis, large hematomas (surgical drainage and debridement) Minor: pain, hematoma	[42]
Brodell, 1995	0.1 ml	NR	Mean: 15, 0.5 weeks	None	CR in 73% at 8 weeks, rest at least some improvement. Recurrence in 14%. NS. (NR)	9.5 ± 1.5 months	Severe events: lymphangitis Minor: mild discomfort	[43]

? unclear, *A *adjustable pressure, *5-ala *5-aminolevulinic acid, *BDSP *betamethasone dipropionate sodium-phosphate, *BoNT-A *botulinum neurotoxin-a, *BTX-A *botulinum toxin type a, *CCT *clinical controlled trial, *CNI *conventional needle injection, *CS *case series, *F *fixed pressure, *FU *follow-up, *5-FU *5-fluorouracil, *GAIS *Global Aesthetic Improvement Scale, *HA *hyaluronic acid, *HDSS *hyperhidrosis disease severity scale, *NAPSI *Nail Psoriasis Severity Index, *no *numbers, *NPT *needle-free jet injection, *NR *not reported, *NS *no significance reported, *OI *overall improvement, *onabotA* onabotulinumtoxinA, *PIH *post-inflammatory hyperpigmentation, *POSAS *patient and observer scar assessment scale, *RCT *randomized controlled trial, *SAL *normal saline, *TCA *triamcinolone acetonide, *TH* triamcinolone hexacetonide, *VSS *Vancouver Scar Scale

**addition to photodynamic therapy

### Alopecia areata

Four studies investigated jet injections to treat alopecia areata (Table 1) [29–32]. Jet injections with betamethasone dipropionate sodium phosphate vs. saline in group A and cyclosporine A vs. saline in group B resulted in hair regrowth in respectively 88.2%, 11.7%, 66.6%, and 16.6% of the patients (4 treatments; no statistical analyses) [29]. Spring-loaded jet injections with TCA resulted in hair regrowth in 62% of the patients (3 treatments; no comparative intervention; no statistical analyses) [30]. TCA with spring-loaded jet injections resulted in hair regrowth in 75% of the patients (3–4 treatments; no comparative intervention; no statistical analyses) [31]. Spring-loaded jet injections with TCA resulted in hair regrowth in 43–49% (≤ 3 treatments; no comparative intervention; no statistical analyses) [32].

### Hyperhidrosis

Four studies investigated a single jet injector treatment for hyperhidrosis (Table 1) [33–36]. Pneumatic powered jet injections compared to needle injections with onabotulinumtoxinA were administered to treat palmar hyperhidrosis and reduced hyperhidrosis disease severity (HDSS) compared to baseline with respectively 1.6 (*p* = 0.031) and 1.25 (*p* = 0.1925) and no significant difference in pain between treatments [33]. Botulinum neurotoxin-A administered with spring-loaded jet injections and needle injections significantly reduced sweat production with respectively 24.7 mg/ml vs. 54.1 mg/ml in palmar and axillar hyperhidrosis compared to baseline (*p* < 0.05*; p* < 0.0001). However, pain was “unacceptable” in half of the patients treated with needle injections and in none of the patients treated with jet injections [34]. Pneumatic jet injections with botulinum neurotoxin-A, resulted in HDSS reduction of 2 and 3 compared to baseline, respectively in patients with axillar and palmoplantar hyperhidrosis (no comparative intervention; *p* < 0.001 in both groups) [35]. Spring-loaded jet injections with botulinum toxin type A resulted in a complete relief of symptoms in 70% of the patients with plantar hyperhidrosis (no comparative intervention; no statistical analyses) [36].

### Nail diseases

Three studies investigated jet injections to treat nail diseases (Table 1) [37–39]. Pneumatic jet injections with TCA were administered periungual to treat nail psoriasis, showing a Nail Psoriasis Severity Index (NAPSI) reduction of 3.7 compared to baseline (4 treatments; no comparative intervention; *p* = 0.0007) [37]. Spring-loaded jet injections with TCA in the posterior nail fold improved nail matrix psoriasis and hyponychial varying from “slight or marked improvement” to “normal nail” in 26% and 90% of the patients respectively (3 treatments; no statistical analyses) [38]. In comparison, the same device with TCA injections in the posterior nail fold showed “matrix improvement” in 73–95%, in psoriasis or lichen planus nails, and “onycholysis improvement” in 47–70% in psoriasis or idiopathic onycholysis nails (≥ 3 treatments, no comparative intervention; no statistical analyses) [39].

### Nonmelanoma skin cancer

Two studies investigated jet injections to treat non-melanoma skin cancer (superficial and nodular BCC, SCC, Bowen’s disease, and Paget’s disease) with 5-aminolevulinic acid (5-ALA) in combination with photodynamic therapy (PDT) (Table 1) [40, 41]. Spring-loaded jet injections with 5-ALA with PDT resulted in an 81% complete response (6 treatments; no comparative intervention; no statistical analyses) [40]. Treatment of PDT with 5-ALA administered with pneumatic injection compared to needle injections resulted in a 77% vs. 65% complete response rate (6 treatments; *p* = 0.012) [41].

### Common warts

Two studies investigated spring-loaded jet injectors to treat palmar and plantar warts (Table 1) [42, 43]. Jet injections with bleomycin resulted in a complete response in 77.5% of the patients (5 treatments; no comparative intervention; no statistical analyses) [42]. Jet injections composed of interferon alfa-n3 resulted in a complete response in 73% of the patients (mean 15 treatments; no comparative intervention; no statistical analyses) [43].

### Other dermatological indications

Four studies investigated jet injections in granuloma annulare, lichen simplex chronicus, psoriasis, and seborrheic dermatitis (Table 2) [44–47]. Spring-loaded jet injection with TCA vs. normal saline resulted in complete response in 68% vs. 44% of the granuloma annulare lesions (2–4 treatments; no statistical analyses) [45]. Spring-loaded jet injections with TCA or placebo showed “excellent” results in respectively 66% vs. 46% of the lichen simplex chronicus patients (8 treatments, *p* = 0.80) [46]. In psoriasis patients, 13.3% of the patients had “better” results with spring-loaded jet injections (Port-O-Jet), 6.7% had “better” results with needle injections and 80% had “equal” results (1 treatment; no statistical analyses) [44]. Spring-loaded jet injections composed of vitamin B6, glycyrrhizin, metronidazole and hyaluronic acid resulted in a mean Investigator Global Assessment (IGA) reduction of 1.2 points, in patients with seborrheic dermatitis (3 treatments; no comparative intervention; *p* < 0.05) [47].

**Table 2:** Characteristics and summary of results of included studies using needle-free jet injectors in other dermatological indications (granuloma annulare, psoriasis, seborrheic dermatitis, local anesthesia, and aesthetics)

**First author, year of publication**	**Dermatological indication**	**Study design**	**No. of patients (+ lesions)**	**Skin type**	**Type jet injector (brand) + (pressure)**	**Pressure in study**	**Fluid**	**Total volume per lesion each treatment**	**Concentration**	**Total no treatments and interval**	**Comparison**	**Results per patient + significance (results per lesion + significance)**	**Follow-up time**		**Adverse reactions**	**Ref**
Sparrow et al. 1975	Granuloma Annulare	CCT	45 (58)	NR	Spring-loaded (Port-O-Jet) (F)	NR	TCA	0.1–0.3 ml	5 mg/ml	Mean: 2–4, 2–8 weeks	SAL	63.6% cleared more with TCA, 36.4% same response with SAL and TCA (CR in 68% and 44% in resp. TCA and SAL. NS)	2–24 months		Severe: none Minor: erythema, atrophy	[45]
Vasistha and Singh 1978	Lichen simplex chronicus	CCT	30 (?)	NR	Spring-loaded (Dermojet) (F)	1420 psi	A: TCA	0.1 ml	10 mg/ml	8, 1 week	B: NPT + distilled water	“Excellent” in 66% in A and 46% in B, *p* = 0.80. (NR)	1 month		Severe: none Minor: hypo- and depigmentation, aggravation of new patches	[46]
Bleeker 1974	Psoriasis	Prospective cohort	18 (?)	NR	A: Spring-loaded (Port-O-Jet) (F)	NR	TCA	Skin < 5 ml Nails: 0.2–0.6 ml	5 mg/ml	1	B: CNI + TCA	13.3% “better” results with NPT, 6.7% “better” results with CNI, and 80% “equal” results. NS. (NR)	Unclear		NR	[44]
Zhang et al. 2020	Seborrheic dermatitis	Retrospective cohort	72 (72)	NR	NR	NR	1: Vitamin B6 2: glycyrrhizin 3: metronida-zol 4: HA	1: 4 ml 2: 20 ml 3: 8 ml 4: 6 ml	1: 50 mg/ml 2: 2 mg/ml 3: 5 mg/ml 4: 0.5 mg/ml	3, 2 weeks	None	Mean IGA 6.79 ± 1.20, 6.28 ± 0.98 and 5.58 ± 0.93 resp. baseline, 4 and 6 weeks. Erythema and hydration improved (*p* < 0.001; *p* < 0.05). Roughness of the skin and lipid level not significant. (NR)	2 weeks		Severe: none Minor: itching	[47]
Saghi et al. 2015	Local anesthesia	RCT	53 (?)	NR	Pneumatic (NR) (NR)	NR	Lidocaine	1 ml	10 mg/ml	1	B: CNI + lidocaine 10 mg/ml	VAS injection: 1.1 ± 1 and 4.4 ± 1.4, resp. in A and B, *p* < 0.0001. No difference in suture pain, *p* > 0.05. (NR)	No FU		NR	[48]
Mumford et al. 1976	Local anesthesia	CCT	82 (NR)	NR	Spring-loaded (Syrijet) (A)	2000 or 2600 psi	Xylocaine	Unclear	20 mg/ml	1	CNI + lidocaine 20 mg/ml	No pain in 94% and 83%, resp. NPT and CNI. Children preferred NPT unanimously. NS. (NR)	No FU		Severe: none Minor: oozing	[49]
Queralt et al. 1995	Local anesthesia	Prospective cohort	168 (206)	NR	Spring-loaded (MadaJet) XL (F)	1800 psi	Mepivacaine chloride	Ca 1.3 ml	10 mg/ml	1	None	NR. (CR in 79.61%, in others minimal discomfort. NS)	None		Severe: none Minor: edema	[50]
Cho et al. 2019	Aesthetic (facelifts)	RCT	10 (20)	III–IV	Pneumatic (SheMax) (A)	52.6 psi	A: Hypertonic glucose	8 ml	200 mg/ml	3, 4 weeks	B: Isotonic glucose 50 mg/ml	NR (Mean overall GAIS in A and B resp. 2.5 ± 0.707 and 3.1 ± 0.876, *P* = 0.005)		3 months	Severe: none Minor: bleeding, redness, worsening	[51]
Choi et al. 2017	Aesthetic (wrinkles)	RCT	24 (24)	III–V	A: Pneumatic (Innojector) (A)	? (level 5)	HA	1.05 ml	NR	3, 2 weeks	B: MNI + HA C: NPT + placebo D: MNI + placebo	WSRS reduction of 1.00 ± 0.63 and 1.50 ± 0.55, resp. A and B in week 16, *p* < 0.05; *p* < 0.01. Reduction in C and D not significant. (NR)	3 months		Severe: none Minor: pain	[52]
Espinoza et al. 2020	Aesthetic (wrinkles)	Retrospective cohort	34 (34)	NR	Pneumatic (AirGent 2.0) (A)	NR	HA	NR (0.09 ml per injection)	NR	Mean: 2.5–3, 12 weeks	None	Mean Lemperle Rating Score decreased 1 degree in all treated areas. NS. (NR)	6 months		Severe: none Minor: bruises, swelling, erythema, scabs	[53]
Cheng et al. 2018	Aesthetic (skin rejuvenation)	Prospective cohort	28 (28)	III–V	Pneumatic (JetPeel-3 V) (A)	103 psi	Non-crosslinked HA	5 ml	NR	5, 1 week	None	“Improved” and “much improved” GAIS score rated by patients and dermatologists in week 5 in resp. 42.86% and 57.14%. NS. (NR)	3 months		Severe: none Minor: none	[54]
Kwon et al. 2018	Aesthetic (facelift/skin rejuvenation)	Prospective cohort	22 (22)	III–IV	Pneumatic (Ultra Beau-jetT) (A)	18.1–72.5 psi	Hypertonic glucose	0.4–6.0 ml	200 mg/ml	1	None	Improvement in 91% post-treatment. NS. (NR)	3 months		Severe: none Minor: erythema, blebs	[55]
Levenberg et al. 2010	Aesthetic (skin rejuvenation)	Prospective cohort	34 (69)	I–IV	Pneumatic (AirGent) (A)	NR	Cross linked HA	NR (mass 2 mg)	NR	1–4, 3–4 weeks	None	80% was (very) satisfied. (Long-term wrinkles reduced 27.6% and 21.2%, resp. face and neck, *p* < 0.05; *p* < 0.05. Long term OI in dorsal hands was good, *p* < 0.05)	1–18 months		Severe: none Minor: bleeding, erythema, edema, tenderness, PIH	[56]

? unclear, *A *adjustable pressure, *BPT *plum-blossom needle injection, *CCT *clinical controlled trial, *CNI *conventional needle injection, *CR *complete response, *CS *case series, *F *fixed pressure, *FU *follow-up, *HA *hyaluronic acid, *GAIS *Global Aesthetic Improvement Scale, *IGA *Investors Global Assessment, *MNI *multi-needle injection, *NPT *needle-free jet injection, *no *numbers, *NR *not reported, *NS *no significance reported, *PIH *post inflammatory hyperpigmentation, *PR *partial response, *RCT *randomized controlled trial, *SAL *normal saline, *TCA *triamcinolone acetonide, *VAS *Visual Analogue Scale, *WSRS *Wrinkle Severity Rating Scale

### Local anesthesia

Three studies investigated local anesthesia administered by a spring-loaded jet injector before suturing or performing dermatological surgery (Table 2) [48–50]. Jet injections with mepivacaine chloride resulted in “no pain” in 79.6% of the lesions during surgery (no comparative intervention; no statistical analyses) [50]. Lidocaine administered with a jet injector compared to injections with a hypodermic needle resulted in a mean anesthesia-related Visual Analogue Scale (VAS) score of 1.1 vs. 4.4 respectively (*p* < 0.0001), while suturing-related pain was not significantly different (*p* > 0.05) [48]. Lidocaine administered with a jet injector vs. needle injections resulted in “no pain” during suturing in respectively 94% vs. 83% of the children [49].

### Aesthetics

Six studies investigated intralesional pneumatic jet injections in the face or neck for aesthetic purposes (Table 2) [51–56]. Jet injections with hypertonic glucose compared to isotonic glucose improved GAIS with a mean score of respectively 2.5 ± 0.7 vs. 3.1 ± 0.9 (3 treatments; *p* = 0.005) [51]. To compare, jet injections with non-crosslinked hyaluronic acid resulted in “improved” and “much improved” GAIS in 42.9% and 57.1% of the patients respectively (5 treatments; no comparative intervention; no statistical analyses) [54]. Crosslinked hyaluronic acid using jet injections reduced mean Fitzpatrick–Goldman Wrinkle Classification with 21.2% and 27.6%, respectively in the neck and face (1–4 treatments; no comparative intervention; *p* < 0.05; *p* < 0.05) [56]. Hyaluronic acid with jet injections or multi-needle injections and placebo with jet injections or multi-needle injections reduced Wrinkle Severity Rating Scale compared to baseline with 1.0 ± 0.6 vs. 1.5 ± 0.6 vs. 0.5 ± 0.8 vs. 0.5 ± 0.6, respectively (3 treatments; *p* < 0.05; *p* < 0.01; *p* > 0.05; *p* > 0.05) [52]. Jet injections with hyaluronic acid reduced Mean Lemperle Rating Score with one point in all areas (2.5 treatments; no comparative intervention; no statistical analyses) [53]. Jet injections with hypertonic glucose showed “slight” or “notable” improvement in 91% of the patients (1 treatment; no comparative intervention; no statistical analyses) [55].

### Adverse reactions

The majority of the adverse reactions were mild and the most common were local erythema, pain, hypo- and hyperpigmentation, bruising, hematoma, atrophy, swelling, and itching (Tables 1 and 2). No serious adverse events were reported. However, two studies that investigated bleomycin or interferon alfa-n2 delivered with a spring-loaded jet injector for palmar and plantar warts reported severe events including cellulitis, lymphangitis, and large hematomas, which needed surgical drainage and debridement [42, 43]. Also, TCA administered by a spring-loaded jet injector for the treatment of alopecia areata resulted in bleeding from the arteria temporalis in one patient, which was controlled by firm pressure [32].

### Methodological quality assessment

**Fig. 2:**
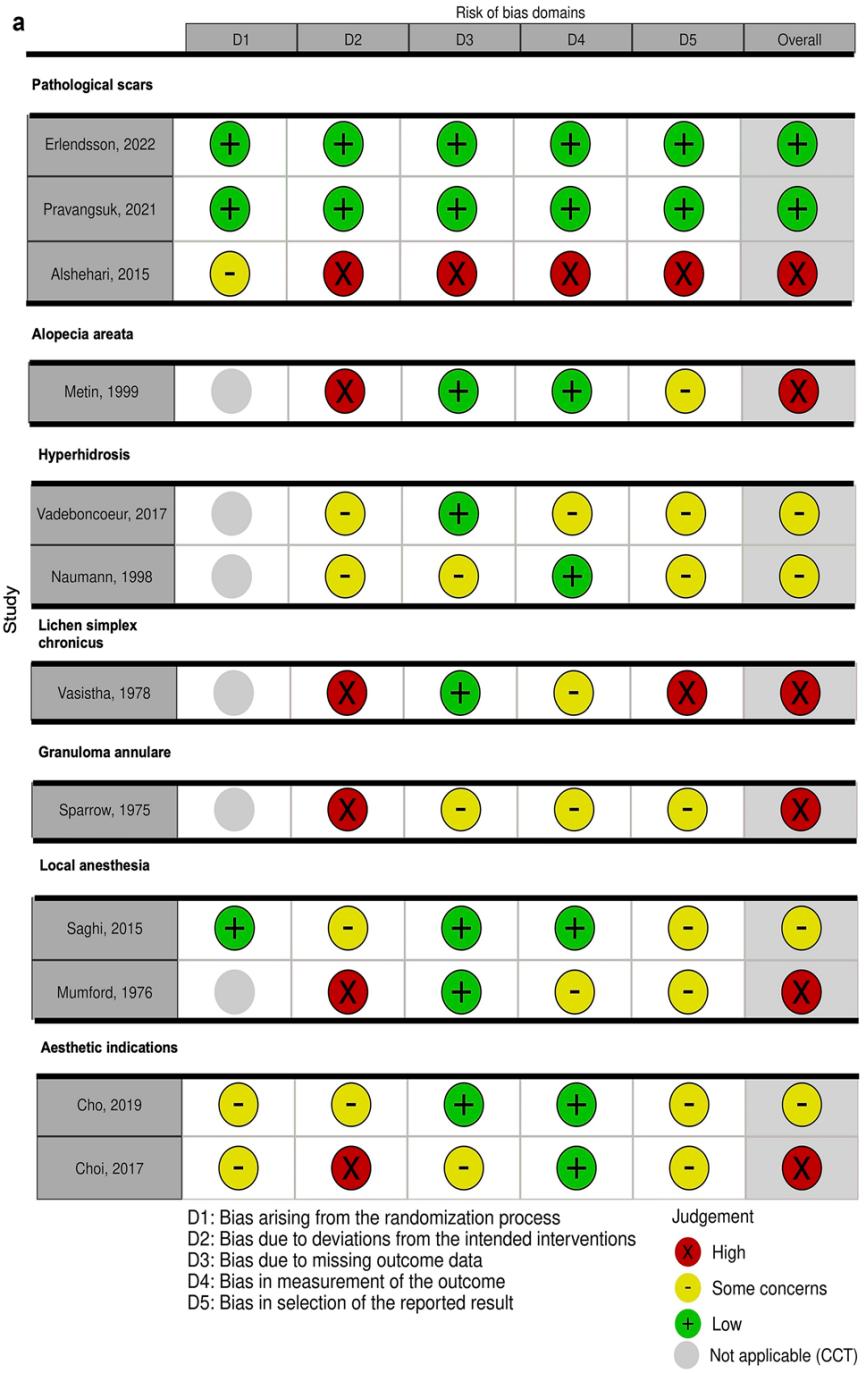

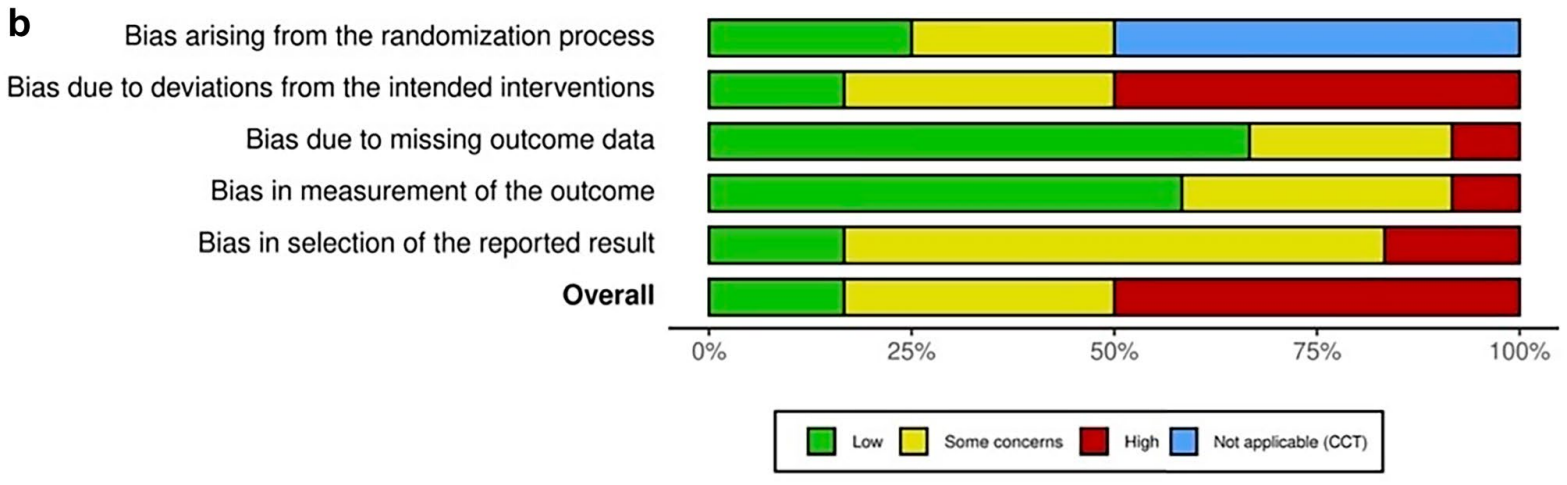
**a** Risk of bias in the included (non) randomized controlled trials was categorized as high, low or some concerns according to the Cochrane risk-of-bias 2.0 assessment tool. Overall, risk of bias was high because of poor methodological quality, particularly in domain 2 and 5. **b** Methodological quality of the (non) randomized controlled trials according to the Cochrane Collaborations risk-of-bias 2.0 tool assessment

**Fig. 3:**
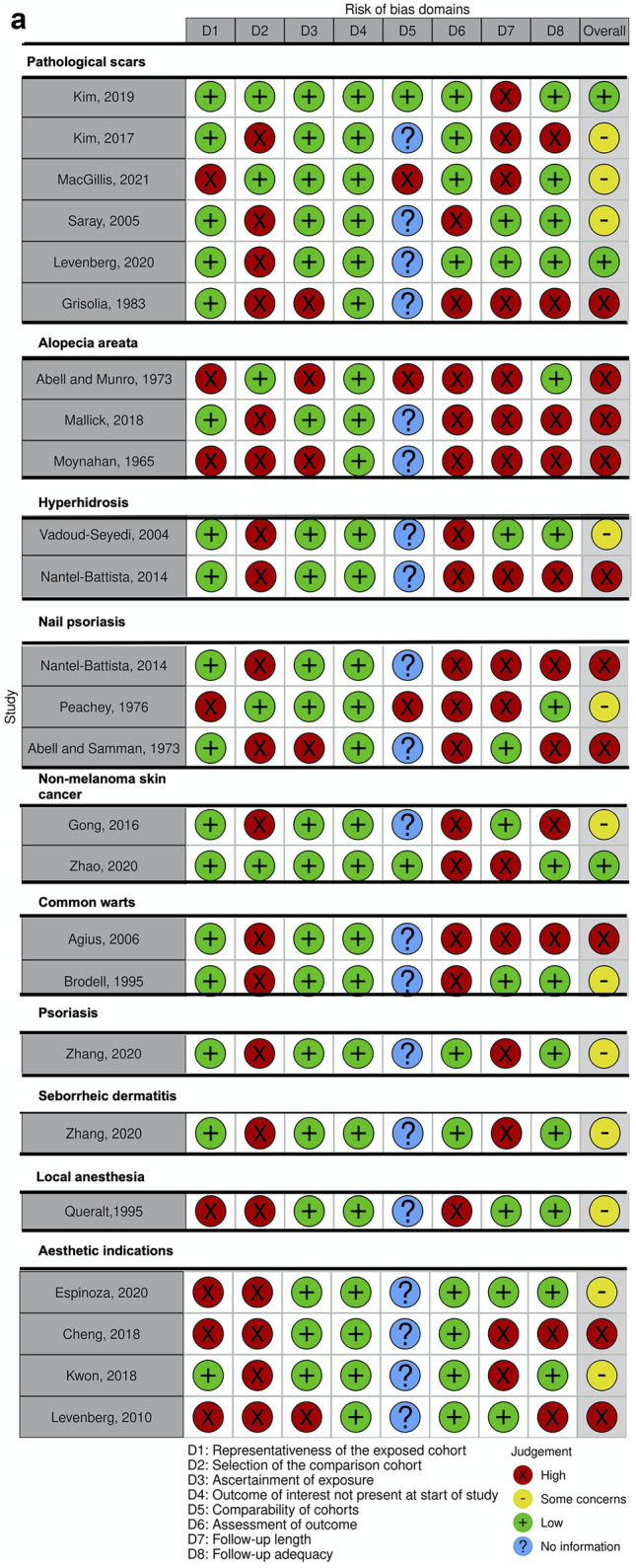

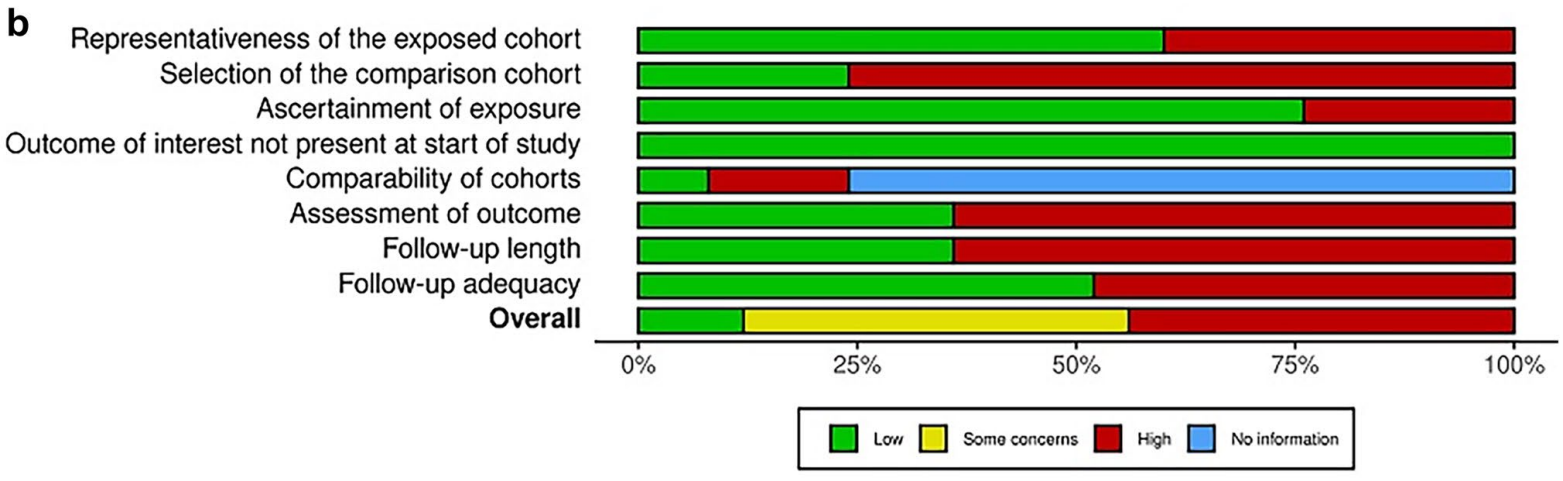
**a** Risk of bias in the included cohort studies and case series was categorized as high, low, some concerns or not applicable according to the Newcastle–Ottawa Scale. Overall, risk of bias was high because of poor methodological quality, particularly in domains 2, 6, and 7. **b** Methodological quality of the included cohort studies and case series according to the Newcastle–Ottawa Scale

Overall risk of bias assessed with Cochrane’s ROB 2.0 tool was “high” in six RCTs and CCTs, “some concerns” in four studies, and “low” in two studies (Fig. 2a). Methodological quality was particularly poor due to deviations from the intended intervention and selection bias (Fig. 2b). According to the Newcastle–Ottawa Scale, overall risk of bias in the included cohorts and case series was “high” in eleven, “some concerns” in another eleven, and “low” in three studies (Fig. 3a) [16]. Methodological quality was particularly poor due to lack of comparative cohorts, lack of blinding, and short follow-up time (Fig. 3b).

## Discussion

In this systematic review, we summarized and critically appraised the current evidence on the efficacy and safety of jet injector-assisted intralesional treatments for dermatological indications. We selected 37 studies including 12 (randomized) controlled trials. The majority of studies had a “high risk of bias” or “some concerns” and only five studies (investigating acne scars, hypertrophic scars, keloids, and non-melanoma skin cancer) had “low risk of bias”. Furthermore, 19 of 37 studies lacked statistical analysis for the reported outcomes.

Due to large heterogeneity among studies with respect to a.o. study design, indication, type of jet injector, therapeutics, and outcome measures, a meta-analysis could not be performed.

Significant favorable effectiveness was reported in 13 of 15 studies, in which statistical analyses were reported. These studies investigated intralesional jet injections in scars, hyperhidrosis, nail psoriasis, non-melanoma skin cancer, seborrheic dermatitis, local anesthesia, and aesthetic indications. Most studies investigated keloids and other types of scars (hypertrophic, atrophic, and burn scars) and showed good efficacy and high tolerability [21–26]. Additionally, our review shows that despite differences in viscosity, several fluids have been successfully administered with jet injectors.

None of the included studies compared the use of spring-loaded vs. pneumatic jet injectors. In studies published before 2000, only spring-loaded jet injectors were used because pneumatic jet injectors were not yet introduced. Importantly, spring-loaded jet injectors were associated with a number of severe adverse reactions, including fluctuating cortisol levels and arteria temporalis damage in alopecia areata treated with TCA. Cellulitis, large hematomas, and lymphangitis occurred in patients with warts treated with spring-loaded devices and bleomycin or interferon alfa-n3 [30, 32, 42, 43]. In contrast, no severe adverse reactions were reported in studies that investigated pneumatic jet injectors. Possibly, this could be related to the tunable settings for pneumatic jet injectors enabling safer and more effective treatment settings based on clinical endpoints, which are not available for spring-loaded injectors [57].

Only five of the included studies compared patient-reported pain between needle-free jet injectors and conventional needle injections [33, 34, 41, 48, 49]. Jet injections with lidocaine caused significantly less injection-related pain, and less procedure-related pain with 5-ALA and PDT treatment in non-melanoma skin cancer compared to needle injections with 5-ALA and PDT [34, 41, 48]. Jet injections with botulinum toxin for palmar and axillar hyperhidrosis and with xylocaine for local anesthesia in children were better tolerated than conventional needle injections; however, no statistical analyses were performed [34, 49]. On the other hand, two studies investigating local anesthesia with lidocaine and palmar hyperhidrosis with onabotulinumtoxinA reported no significant difference in procedure-related pain between jet injections and conventional needle injections [33, 48].

Risk of bias assessment resulted in two high-quality RCTs. The results of these studies suggest that jet injections with 5-FU and TCA and jet injections with saline in atrophic acne scars (boxcar and rolling) are efficacious, safe, and well-tolerated [20, 22]. Also, favorable efficacy and safety were found in cohort studies with low risk of bias for intralesional jet injections with 5-FU combined with corticosteroids in keloids and with hyaluronic acid in atrophic acne scars.

To our knowledge, this is the first systematic review that evaluated the efficacy and safety of intralesional treatment with jet injectors for dermatological indications. The strengths of this study include the use of a comprehensive database search, reporting of outcome measures as efficacy and adverse reactions, addressing jet injector settings, critical methodological quality assessment, and inclusion of all study designs with no limitation to publication date. Limitations of this systematic review include a majority of studies in cohorts or case series, noncomparative studies, poor methodological quality of the included studies, and missing of important clinical data such as skin type.

At our tertiary outpatient clinic, patients with keloids, hypertrophic scars, and recalcitrant warts are commonly treated with spring-loaded or pneumatic injectors to administer TCA, bleomycin or a mixture of both.

Moreover, we believe there is a significant clinical benefit of jet injector treatment in children (e.g., for keloids and hypertrophic scars), because in our experience they tolerate the jet injections much better and cause less anxiety than conventional hypodermic needle injections.

Importantly, we strongly recommend the use of protective safety measures such as smoke evacuators and face masks due to the potential formation of harmful aerosols, especially when antineoplastic drugs such as bleomycin or 5-FU are administered [9]. Moreover, caution should be taken when using spring-loaded jet injectors in anatomical areas around large vessels, nerves, and bone, because potential damage can be inflicted with this type of fixed-setting jet injectors [32].

Contemporary deficiencies of modern jet injectors include drug spill (residual fluid on the skin surface and formation of potentially harmful airborne small-droplet aerosols). Also, gas-compressed energy-based jet injectors create a relatively loud noise during the injection phase which may lead to anxiety in some patients [6, 12, 58, 59]. Therefore, opportunities for improvement of the needle-free injection technology in the future will lie in optimizing the injection efficiency, creating less noisy (smaller) devices, and the development of new technology to reduce the production or capture potentially harmful aerosols. Moreover, with respect to future research, good quality RCTs investigating the efficacy and safety of jet injectors in dermatology are highly needed to conduct a meta-analysis and produce stronger evidence that can be used to provide solid evidence-based recommendations for the use of jet injectors in clinical practice.

In conclusion, this systematic review presents an overview and methodological quality assessment of clinical data on the efficacy and safety of intralesional jet injection treatments for dermatological indications. Limited good quality data suggest that intralesional jet injection treatments with 5-FU and TCA in hypertrophic scars and with saline in atrophic acne scars are efficacious and well-tolerated [20, 22]. In addition, some evidence suggests that jet injector treatment might be less painful for patients than conventional needle injections for certain indications. More high-quality randomized controlled trials are needed to provide future evidence-based recommendations for clinical practice.

## Data Availability

PRISMA checklist, electronic literature search, figures, and in- and excluded studies can be found as attachments. Other datasets generated during and/or analyzed during the review are available from the corresponding author on request.

## Abbreviations

5-ALA: 5-Aminolevulinic acid
BCC: Basal cell carcinoma
CCTs: Controlled clinical trial
DCJIs: Disposable cartridge jet injectors
GAIS: Global Aesthetic Improvement Scale
HDSS: Hyperhidrosis disease severity scale
MB: Morbus Bowen
MUNJIs: Multi-use nozzle jet injectors
NAPSI: Nail Psoriasis Severity Index
NOS: Newcastle Ottawa Scale
PDT: Photodynamic therapy
PRISMA: Preferred Reporting Items for Systematic Reviews and Meta-analyses
PROSPERO: International Prospective Register of Systematic Reviews
RCTs: Randomized controlled trial
ROB: Risk of bias tool
Robvis: Risk of bias visualization
SCC: Squamous cell carcinoma
TCA: Triamcinolone acetonide

## References

[1] Benson HA (2005). Transdermal drug delivery: penetration enhancement techniques, (in eng). Curr Drug Deliv.

[2] Prausnitz MR, Langer R. Transdermal drug delivery (in eng). Nat Biotechnol. 2008;26(11):1261-8. 10.1038/nbt.1504[pii]. 10.1038/nbt.1504 PMC2700785 18997767

[3] Ita K. Perspectives on Transdermal Electroporation (in eng). Pharmaceutics, 2016;8(1). 10.3390/pharmaceutics8010009[pii]. 10.3390/pharmaceutics8010009 PMC4810085 26999191

[4] Schramm J, Mitragotri S (2002). Transdermal drug delivery by jet injectors: energetics of jet formation and penetration, (in eng). Pharm Res.

[5] Szunerits S, Boukherroub R (2018). Heat: a highly efficient skin enhancer for transdermal drug delivery, (in eng). Front Bioeng Biotechnol.

[6] Barolet D, Benohanian A (2018). Current trends in needle-free jet injection: an update, (in eng). Clin Cosmet Investig Dermatol.

[7] Mitragotri S. Current status and future prospects of needle-free liquid jet injectors (in eng). Nat Rev Drug Discov. 2006;5(7):543-8. 10.1038/nrd2076[pii]. 10.1038/nrd2076 16816837

[8] Chobert B et al. Injections intra-dermiques et transcutanées sans aiguille. Présentation d’un appareil. Bulletin de l'Académie Vétérinaire de France. 1960;507–512. [Online]. Available: https://www.persee.fr/doc/bavf_0001-4192_1960_num_113_9_4141.

[9] Bik L, van Doorn MBA, Biskup E, Ortner VK, Haedersdal M, Olesen UH (2021). Electronic pneumatic injection-assisted dermal drug delivery visualized by ex vivo confocal microscopy, (in eng). Lasers Surg Med.

[10] Schoppink J, Fernandez Rivas D. Current engineering and clinical aspects of needle-free injectors: a review. Adv Drug Deliv Rev. 2022;182:114109. 10.1016/j.addr.2021.114109. 10.1016/j.addr.2021.114109 34998902

[11] Taddio A et al. Survey of the prevalence of immunization non-compliance due to needle fears in children and adults (in eng). Vaccine. 2012;30(32):4807-12. 10.1016/j.vaccine.2012.05.011[pii]. 10.1016/j.vaccine.2012.05.011 22617633

[12] Hogan NC, Taberner AJ, Jones LA, Hunter IW (2015). Needle-free delivery of macromolecules through the skin using controllable jet injectors, (in eng). Expert Opin Drug Deliv.

[13] Logomasini MA, Stout RR, Marcinkoski R. Jet injection devices for the needle-free administration of compounds, vaccines, and other agents (in eng). Int J Pharm Compd. 2013;17(4):270–80. Available: https://www.ncbi.nlm.nih.gov/pubmed/24261141. 24261141

[14] Kale TR. Needle free injection technology - an overview. Innov Pharm. 2014;5(1). 10.24926/IIP.V5I1.330.

[15] Page MJ et al. The PRISMA 2020 statement: an updated guideline for reporting systematic reviews (in eng). BMJ. 2021;372(n71) Mar 29 2021. 10.1136/bmj.n71. 10.1136/bmj.n71 PMC8005924 33782057

[16] Wells GA, Shea B, O’Connell D, Peterson J, Welch V, Losos M, Tugwell P. The Newcastle-Ottawa Scale (NOS) for assessing the quality of nonrandomised studies in meta-analyses. 2021. http://www.ohri.ca/programs/clinical_epidemiology/oxford.asp. Accessed 19 Aug 2021.

[17] McGuinness LA, Higgins JPT (2021). Risk-of-bias VISualization (robvis): an R package and Shiny web app for visualizing risk-of-bias assessments, (in eng). Res Synth Methods.

[18] Montagnon CM, et al. Pyoderma gangrenosum in hematologic malignancies: a systematic review (in eng). J Am Acad Dermatol. 2020;82(6):1346-1359. 10.1016/j.jaad.2019.09.032[pii]. 10.1016/j.jaad.2019.09.032 31560977

[19] van Winden ME, et al. Effectiveness and safety of systemic therapy for psoriasis in older adults: a systematic review (in eng). JAMA Dermatol. 2020;156(11):1229-1239. 10.1001/jamadermatol.2020.2311[pii]. 10.1001/jamadermatol.2020.2311 32822455

[20] Erlendsson AM, et al. A one-time pneumatic jet-injection of 5-fluorouracil and triamcinolone acetonide for treatment of hypertrophic scars-a blinded randomized controlled trial (in eng). Lasers Surg Med. 2022. 10.1002/lsm.23529. 10.1002/lsm.23529 35266202

[21] Alshehari A, Wahdan W, Maamoun MI (2015). Comparative study between intralesional steroid injection and silicone sheet versus silicone sheet alone in the treatment of pathologic scars.

[22] Pravangsuk J, Udompataikul M, Cheyasak N, Kamanamool N. Comparison of normal saline injection with pneumatic injector to subcision for the treatment of atrophic acne scars (in eng). J Clin Aesthet Dermatol. 2021;14(5):50–55. Available: https://www.ncbi.nlm.nih.gov/pubmed/34188750. PMC8211335 34188750

[23] Kim BY, Chun SH, Park JH, Ryu SI, Kim IH (2019). Prospective evaluation of atrophic acne scars on the face with needle-free high-pressure pneumatic injection: quantitative volumetric scar improvement, (in eng). Dermatol Surg.

[24] Kim H, Yoo KH, Zheng Z, Cho SB (2017). Pressure- and dose-controlled transcutaneous pneumatic injection of hypertonic glucose solution for the treatment of atrophic skin disorders, (in eng). J Cosmet Laser Ther.

[25] MacGillis D, Vinshtok Y (2021). High-velocity pneumatic injection of non-crosslinked hyaluronic acid for skin regeneration and scar remodeling: a retrospective analysis of 115 patients, (in eng). J Cosmet Dermatol.

[26] Saray Y, Güleç AT. Treatment of keloids and hypertrophic scars with dermojet injections of bleomycin: a preliminary study (in eng). Int J Dermatol. 2005;44(9):777-84. 10.1111/j.1365-4632.2005.02633.x[pii]. 10.1111/j.1365-4632.2005.02633.x 16135153

[27] Levenberg A, Vinshtok Y, Artzi O (2020). Potentials for implementing pressure-controlled jet injection in management of keloids with intralesional 5FU and corticosteroids, (in eng). J Cosmet Dermatol.

[28] Grisolia GA, Danti DA, Santoro S, Panozzo G, Bonini G, Pampaloni A (1983). Injection therapy with triamcinolone hexacetonide in the treatment of burn scars in infancy: results of 44 cases, (in eng). Burns Incl Therm Inj.

[29] Metin A, Delice I, Subasi S. Treatment of alopecia areata with intralesional jet injection of bethamethasone or Cyclosporine A, presented at the 8th Congr EADV. 1999.

[30] Abell E, Munro DD (1973). Intralesional treatment of alopecia areata with triamcinolone acetonide by jet injector, (in eng). Br J Dermatol.

[31] Mallick YA, Kapadia NF, Mansoor M, Talat H. Efficacy of intralesional triamcinolone acetonide in alopecia areata by Dermojet at Abbasi Shaheed Hospital, Karachi, Pakistan. Rawal Med J. 2018;43(2):227–230. Available: http://inis.iaea.org/search/search.aspx?orig_q=RN:49055153.

[32] Moynahan EJ, Bowyer A (1965). Development of jet injection and its application to intralesional therapy in dermatology, (in eng). Br Med J.

[33] Vadeboncoeur S, Richer V, Nantel-Battista M, Benohanian A (2017). Treatment of palmar hyperhidrosis with needle injection versus low-pressure needle-free jet injection of onabotulinumtoxinA: an open-label prospective study, (in eng). Dermatol Surg.

[34] Naumann M, Bergmann I, Hofmann U, Hamm H, Reiners K (1998). Botulinum toxin for focal hyperhidrosis: technical considerations and improvements in application, (in eng). Br J Dermatol.

[35] Kim HM, Lee MJ, Lee MH, Lee H (2020). Pressure-and dose-controlled, needle-free, transcutaneous pneumatic injection of botulinum neurotoxin-a for the treatment of primary axillary and palmoplantar hyperhidrosis, (in eng). Skin Res Technol.

[36] Vadoud‐Seyedi J. Treatment of plantar hyperhidrosis with botulinum toxin type A (in eng). Int J Dermatol. 2004;43(12):969-71. 10.1111/j.1365-4632.2004.02304.x[pii]. 10.1111/j.1365-4632.2004.02304.x 15569036

[37] Nantel-Battista M, Richer V, Marcil I, Benohanian A. Treatment of nail psoriasis with intralesional triamcinolone acetonide using a needle-free jet injector: a prospective trial, (in eng). J Cutan Med Surg. 2014;18(1):38–42. 10.2310/7750.2013.13078. 10.2310/7750.2013.13078 24377472

[38] Peachey RD, Pye RJ, Harman RR (1976). The treatment of psoriatic nail dystrophy with intradermal steroid injections, (in eng). Br J Dermatol.

[39] Abell E, Samman PD (1973). Intradermal triamcinolone treatment of nail dystrophies, (in eng). Br J Dermatol.

[40] Gong Y (2016). Needle-free injection of 5-aminolevulinic acid in photodynamic therapy for the treatment of non-melanoma skin cancer, (in eng). Dermatol Ther.

[41] Zhao W, Wang J, Zhang Y, Zheng B. A retrospective study comparing different injection approaches of 5-aminolevulinic acid in patients with non-melanoma skin cancer (in eng). J Dermatolog Treat. 2020;1–8. 10.1080/09546634.2020.1832186. 10.1080/09546634.2020.1832186 33016837

[42] Agius E, Mooney JM, Bezzina AC, Yu RC. Dermojet delivery of bleomycin for the treatment of recalcitrant plantar warts (in eng). J Dermatolog Treat. 2006;17(2):112-6. 10.1080/09546630600621987[pii]. 10.1080/09546630600621987 16766336

[43] Brodell RT, Bredle DL. The treatment of palmar and plantar warts using natural alpha interferon and a needleless injector (in eng). Dermatol Surg. 1995;21(3):213-8. 10.1111/j.1524-4725.1995.tb00155.x[pii]. 10.1111/j.1524-4725.1995.tb00155.x 7712088

[44] Bleeker JJ (1974). Intralesional triamcinolone acetonide using the Port-O-Jet and needle injections in localized dermatoses, (in eng). Br J Dermatol.

[45] Sparrow G, Abell E (1975). Granuloma annulare and necrobiosis lipoidica treated by jet injector, (in eng). Br J Dermatol.

[46] Vasistha LK, Singh G (1978). Neurodermatitis and intralesional steroids, (in eng). Dermatologica.

[47] Zhang X, et al. Clinical evaluation of sequential transdermal delivery of vitamin B6, compound glycyrrhizin, metronidazole, and hyaluronic acid using needle-free liquid jet in facial seborrheic dermatitis (in eng). Front Med (Lausanne). 2020;7:555824. 10.3389/fmed.2020.555824. 10.3389/fmed.2020.555824 PMC7662080 33195305

[48] Saghi B, Momeni M, Saeedi M, Ghane M. Efficacy of the jet injector in local anaesthesia for small wound sutures: a randomised clinical trial compared with the needle infiltration technique (in eng). Emerg Med J. 2015;32(6):478–80. 10.1136/emermed-2013-203135[pii]. 10.1136/emermed-2013-203135 25052218

[49] Mumford DM, Jackson PL (1976). The successful use of jet anesthetic injections with childhood lacerations, (in eng). Clin Pediatr (Phila).

[50] Queralt CB, Comet Jr V, Cruz JM, Val-Carreres CA. Local anesthesia by jet-injection device in minor dermatologic surgery (in eng). Dermatol Surg. 1995;21(7):649-51. 10.1111/j.1524-4725.1995.tb00524.x[pii]. 10.1111/j.1524-4725.1995.tb00524.x 7606381

[51] Cho SB, Zheng Z, Yoo KH, Kim HJ, Kim H (2019). Split-face comparison study of transcutaneous pneumatic injection therapy with isotonic and hypertonic glucose solutions, (in eng). J Cosmet Dermatol.

[52] Choi SY, Seok J, Kwon HJ, Kwon TR, Kim BJ (2017). Hyaluronic acid injection via a pneumatic microjet device to improve forehead wrinkles, (in eng). J Eur Acad Dermatol Venereol.

[53] Espinoza L, Vinshtok Y, McCreesh J, Tyson J, McSorley M (2020). Kinetic energy-assisted delivery of hyaluronic acid for skin remodeling in middle and lower face, (in eng). J Cosmet Dermatol.

[54] Cheng HY, Chen YX, Wang MF, Zhao JY, Li LF. Evaluation of changes in skin biophysical parameters and appearance after pneumatic injections of non-cross-linked hyaluronic acid in the face (in eng). J Cosmet Laser Ther. 2018;20(7–8):454–461. 10.1080/14764172.2018.1427868. 10.1080/14764172.2018.1427868 29543523

[55] Kwon HH, Choi SC, Park KH, Jung JY (2018). A novel combination regimen with intense focused ultrasound and pressure- and dose-controlled transcutaneous pneumatic injection of hypertonic glucose solution for lifting and tightening of the aging face, (in eng). J Cosmet Dermatol.

[56] Levenberg A, Halachmi S, Arad-Cohen A, Ad-El D, Cassuto D, Lapidoth M (2010). Clinical results of skin remodeling using a novel pneumatic technology, (in eng). Int J Dermatol.

[57] Bik L (2022). Clinical endpoints of needle-free jet injector treatment: an in depth understanding of immediate skin responses, (in eng). Lasers Surg Med.

[58] Bik L, Sangers T, Greveling K, Prens E, Haedersdal M, van Doorn M. Efficacy and tolerability of intralesional bleomycin in dermatology: a systematic review (in eng). J Am Acad Dermatol. 2020;83(3):888-903. 10.1016/j.jaad.2020.02.018[pii]. 10.1016/j.jaad.2020.02.018 32068046

[59] Simmons JA, et al. Characterization of skin blebs from intradermal jet injection: ex-vivo studies, (in eng). J Control Release. 2019;307:200-210. 10.1016/j.jconrel.2019.06.032[pii]. 10.1016/j.jconrel.2019.06.032 31252035

